# Determinants and spatial distribution of institutional delivery in Ethiopia: evidence from Ethiopian Mini Demographic and Health Surveys 2019

**DOI:** 10.1186/s13690-022-00825-2

**Published:** 2022-02-21

**Authors:** Girma Gilano, Samuel Hailegebreal, Biniyam Tariku Seboka

**Affiliations:** 1 Department of Health Informatics, School of Public Health, College of Medicine and Health Sciences, Arba Minch University, Arba Minch, Ethiopia; 2 Department of Health Informatics, School of Public Health, College of Medicine and Health Sciences, Dilla University, Dilla, Ethiopia

**Keywords:** Institutional delivery, Spatial distribution, Ethiopia, EMDHS data

## Abstract

**Background:**

Over the past few decades, maternal and child mortality had drawn the attention of governments and policymakers. Institutional delivery has been among the implementations needed to reduce maternal and child mortality. The fact that the problem was persisted intensified studies to research for more factors. Thus, the current study was intended for further analyses of EMDHS to identify the magnitude, spatial patterns, and predictors of institutional delivery.

**Methods:**

A cross-sectional survey data from EMDHS 2019 was analyzed involving 5488 reproductive-age women regarding institutional deliveries. We presented descriptive statistics using mean, standard deviations, and proportions. To check the nature of the distribution of institutional delivery, we applied the global Moran’s I statistics. Getis-Ord Gi statistics was applied to detect spatial locations, and we applied spatial interpolation to predict unknown locations of institutional delivery using the Ordinary Kriging method. Kulldorff’s SatScan was also applied to identify the specific local clustering nature of institutional delivery using the Bernoulli method. We applied multilevel binary logistic regression for the scrutiny of individual and community-level factors. We applied *P* < 0.25 to include variables in the model and *P* < 0.05 to declare associations. AOR with 95% CI was used to describe variables.

**Results:**

The prevalence of institution/facility delivery was 2666.45(48.58%) in the survey. The average number of children was 4.03 ± 2.47, and most women in this survey were in the age range of the 25-29 years (31.84%) and 30–34 years (21.61%). Women who learned primary education (AOR = 1.52; 95% CI 1.20–1.95), secondary education (AOR = 1.77; 95% CI 1.03–3.07), and higher education (AOR = 5.41; 95% 1.91–15.25), while those who can read and write sentences (AOR = 1.94; 95% 1.28–2.94), Rich (AOR = 2.40 95% CI 1.82–3.16), and those followed 1–2 ANC (AOR = 2.08; 95% CI 1.57–2.76), 3 ANCs (AOR = 3.24; 95% CI 2.51–418), and ≥ 4 ANCs (AOR = 4.91; 95% CI 3.93–6.15) had higher odds of delivering at health institutions.

**Conclusion:**

The institutional delivery was unsatisfactory in Ethiopia, and there were various factors associated differently across the different regions. Pastoralist regions showed high home delivery than institutions which invites further interventions specific to those regions. Factors like age, highest education level achieved, preceding birth interval, literacy status, wealth status, birth order, regions, and rural residences were all affected institutional delivery so that interventions considering awareness, access, and availability of the services are vital.

## Plain English summary

Maternal and child mortality had drawn the attention of governments and policymakers internationally since 1990s. A lot has been said and tried to reduce maternal and child mortality rates by the government of Ethiopia. Initially, toward the end of MDGs period, the country has been successful in achieving some of the goals related to child and maternal mortality. However, after scene of many implementations, now everything looks downcast again. This fact sparked the need to re-assess the status of the country and provide information for further policy decisions. Currently, we were aimed at providing country representative information from EMDHS data regarding the magnitude, spatial pattern, and predictors of in institutional delivery.

Factors like age, highest education level achieved, preceding birth interval, literacy status, wealth status, birth order, regions, and rural residences were all affected institutional delivery. However, since home delivery was higher and more worsen in pastoralist regions in the country, it should draw more attentions to make interventions considering awareness, access, and availability of the services in vital areas.

## Background

The safety of the mother and newborn is always the target during delivery and can be improved by institutional delivery. Developing countries have many constraints in reducing maternal and newborn problems due to access, availability, and awareness [1]. As a country in the Sub-Saharan region, Ethiopia got similar limitations regarding maternal and child cares. Henceforth, the country planned to increase institutional delivery from 20% in 2011 to 60% in 2015 [2]. Because of the various factors, the country wasn’t able to achieve as planned. Many studies indicated that there was a high proportion of home delivery in the country [3–9]. There were various factors cited affecting women to prefer home over institutions for delivery. A study conducted in the Farta district indicated that the magnitude of institutional delivery was 64.4%. Family size, accessibility of transportation, planned pregnancy, information about the place of delivery, participation of women in monthly health conference (PWMHC), information about exempted service, and having antenatal care (ANC) follow up during their last pregnancy were predictors [10, 11]. In other studies, institutional delivery was 38.9% and influenced by focused antenatal care, multiple gestations, urban residence, and formal education of the women [12, 13].

Residence, region, maternal education, wealth status, ANC visit, preceding birth interval, and community media exposure were all predictors of the institutional delivery from other studies [14–16]. Institutional delivery was 18.2% in Oromia regional state, where urban residence, maternal education, pregnancy-related health problems, previous history of prolonged labor, and the decision made by the husbands or relatives put forth a positive correlation [17]. There was high variation throughout different administrative levels in the country. For instance, institutional delivery was 72% in Bule Hora in Oromiya [18]; 89.1% in Damot district in SNNP [19]; 61.5% in North-Western part of the country [20]; 76% in Southwest Ethiopia [21]; 71.7% in Dejen Woreda; 78.8% in Bahir Dar [22]; 18.3% in Dangila woreda; 47% in semi-urban parts of the country [23]; 38% in Mandura district [24]; 53% in Hossana town [25]; 74% in Dallocha town [26] and 49% in Hetosa district [27]. The rate of increase was 5.4% in 2000 and 11.8%,2011 [28]. Ethiopia showed a good achievement toward the millennium development goal five [29], although the contextual facts look unchanged significantly. The plan to upsurge institutional delivery from 20% in 2011 to 60% in 2015 was not adequately achieved until recently from the above literature. In the countries like Ethiopia where resources are very limited, there were limited chances to collect country representative data; however, analyzing the data from EMDHS-2019 enabled us to provide the country-level figures. And it is paramount to identify modified and persistent factors for unsuccessful for the next health plans. Therefore, our study was aimed at identifying the prevalence and factors through spatial, descriptive, and multilevel analyses to support further policies.

## Methods

### Data source and participants

Ethiopia is the country located at (3^o^-14^o^N, 33^o^ – 48°E). The country had undertaken four standard Demographic Health Surveys (EDHS). The country started EDHS in the year 2000 and conducted every five years since then. There were also two Ethiopian Mini Demographic Health Surveys (EMDHS) conducted in 2014 and 2019. EMDHS usually conduct between the standard EDHS (two to three years) after the EDHS conducted. The 2019 EMDHS is the second nationwide mini survey conducted in the country. In Ethiopian DHS, data has been collected using a two-level multistage stratified cluster sampling to pick eligible respondents from rural and urban areas. For the current analysis, we used Ethiopia Mini Demographic Health Survey (EMDHS) 2019 data. All nine regions and two city administrations were involved in the data collection. The regions were further categorized as agrarian (Benishangul-Gumuz Amhara, Southern Nations, Nationalities, and People Gambela, Oromia, Harari, Region (SNNPR), and Tigray), pastoralists (Afar and Somali), and city administrations (Addis Ababa and Dire-Dawa) contextually. We retrieved the data from the DHS website: (www.dhsprogram.com) after the measure program allowed us to download the datasets. The weighted sample became 5488 women who had live births in the last five years before the survey. They conducted the interview on the permanent residents and visitors who stayed the day before the survey in the residences, and it was a face-to-face manner [30].

### Study variables

The outcome variable for this study was the health institutions/facilities delivery, which was coded as “0” if the women gave birth at home and “1” if the women gave birth at a health facility. Institutions/facilities delivery was stated as the births at health institution/facility within five years afore the survey.

#### Individual-level (covariates) variable

Maternal education, maternal age, religion, ANC follow-up, sex of household head, literacy, the total number of children, birth order, preceding birth interval, the timing of 1st ANC visit, wealth index, and marital status were the variables.

#### Community-level variable

Region and place of residence.

### Statistical analysis

#### Descriptive statistics

Before conducting the descriptive data analysis, we weighted the data to adjust the non-proportional allocation of samples to strata and regions. Then, descriptive statistics were presented using weighted and unweighted frequencies, mean ± (standard deviations), and percentage, while all analyses were performed using STATA version 15 (STATA Corporation. IC., TX, USA). The mean-variance inflation factor also was checked to be 3.53, which was in the acceptable range.

#### Spatial analysis

For spatial analysis, we used ArcGIS 10.7 that determined the clustering, dispersion, and random distribution nature of the institutional delivery. Moran’s I output lies between (− 1 to + 1). The values close to − 1 indicated dispersed institutional delivery, and those closes to + 1 indicated clustering distribution. After discovering significant global autocorrelation, we tested the local Getis Ord statistics to identify the areas with high and low institutional deliveries [31].

#### Spatial interpolation

For statistical optimization of the weight, the Ordinary Kriging spatial interpolation method was applied, and enabled us to make the prediction of institutional delivery for un-sampled areas of the country.

#### SaTscan analysis

SaTScan Version 9.6 software was used for the local cluster detection. A circular window that moves systematically throughout the study area was used to identify a significant clustering of institutional delivery. We presented the results of primary and secondary observed clusters using log-likelihood (LL) and *p*-value < 0.05.

#### Multilevel binary logistic regression

Since the data from country representative surveys are usually clustered or have hierarchical structure, we applied multilevel analysis. We went through four consecutive models building strategies to identify felicitous predictors of institutional delivery in the country. Model 0 is an empty/null (the intercept only model) existed before addition of the predictors. Model 1 (fixed effect model) included all individual-level variables that were initially significant at *p*-value of < 0.25 to determine the level of variance explained by the model. Model 2 (random effect model) included cluster-level (community -level) variables and model 3 (the mixed effect model) was the final model in which both the individual and community level variables introduced to judge final model performance. The log of the probability of the institutional delivery was modeled using multilevel binary logistic regression as:

$\log \left(\frac{\uppi_{\mathrm{ij}}}{1-{\uppi}_{\mathrm{ij}}}\right)={\beta}_{0}+{\beta}_{0}{\mathrm{X}}_{\mathrm{ij}}+{\beta}_{2}{\mathrm{Z}}_{\mathrm{ij}}+uij$; where, i and j are the level 1 (individual) and level 2 (community) units; X and Z refer to individual and community-level variables, in sequence. Πij is the probability of the institutional delivery for the i^th^ mother in the j^th^ community. We resolute random effect using Intra-community Correlation (ICC), $\mathrm{ICC}=\frac{{\upsigma^2}_a}{{\upsigma^2}_a+{\upsigma^2}_b}$; where, σ^2^_*a*_ is the community level variance and σ^2^_*b*_ indicates individual level variance. The variance (σ^2^_*b*_) is equal to ${\pi}^2\left/ 3\right.$ which is the fixed value. Likelihood Ratio (LR) test for model comparison and deviance (−2LL) for the goodness of fit check were calculated, while Median Odds Ratio (MOR) and Proportional Change in Variance (PCV) were also estimated [32].

Finally, the mixed effect model, which included both fixed and random effect variables were fitted. To include variable in the model *p*-value < 0.25 and to declare association *p*-value< 0.05 were used.

AOR with 95% CI was also used to articulate the results.

## Results

### Descriptive statistics

#### Institutional delivery

We analyzed the data on 5488 weighted reproductive-age women who gave birth in the last five years earlier to the survey and found 2666.45(48.58%) of them delivered at health institution/facility.

##### Individual-level characteristics

The average number of children in families in the survey was 4.03 ± 2.47, and the average preceding birth interval was 40.61 ± 26.30 months. Most women in this survey were in the age group of the 25-29 years (31.84%) and 30–34 years (21.61%). In other words, 53.55 and 35.47% were learned no education and primary education, respectively. Similarly, 47.45% of the women had no ANC follow-ups, while 30.52% of women were followed ANC of ≥4 visits. Economically, 45.53% of the women were poor, while 35.57% were in the rich category. Nearly two-thirds of the women cannot read and write (64.34%), and those who can read or write the complete sentence were 20.83%.

##### Community-level characteristics

The higher portions of the women were from Oromia (40.17%), SNNP (19.94%), and Amhara (18.82%) regions. Three-fourth (75.20%) of the women were from rural-based places of residences (Table 1).

**Table 1:** Socio-demographic characteristics of women aged 14–49 year in Ethiopia, EMDHS 2019

Variables	Unweighted frequency (%)	Weighted frequency (%)
**Age in 5 yrs group**		
15–19	295 (5.17)	263.25 (4.80)
20–24	1131 (19.82)	1017.74 (18.54)
25–29	1847 (32.37)	1747.6 (31.84)
30–34	1227 (21.50)	1185.75 (21.61)
35–39	757 (13.27)	797.16 (14.56)
40–44	112 (1.96)	368.35 (6.69)
45–49		107.38 (1.96)
**Region**		
Tigray	453 (7.94)	370.60 (6.75)
Afar	650 (11.39)	85.56 (1.56)
Amhara	503 (8.82)	1032 (18.82)
Oromia	717 (12.57)	2204.46 (40.17))
Somali	636 (11.15)	408.10 (7.44)
Benishangul	526 (9.22)	66.89 (1.22)
SNNPR	651 (11.41)	1105.91 (19.94)
Gambela	436 (7.64)	23.85 (0.43)
Harari	445 (7.80)	16.28 (0.30)
Addis Ababa	290 (5.08)	155.73 (2.84)
Dire Dawa	399 (6.99)	29.56 (0.54)
**Highest educational level**		
No education	3125 (54.77)	2938.85 (53.55)
Primary	1810 (31.72)	1946.96 (35.47)
Secondary & Higher	473 (8.29)	408.61 (7.45)
Above higher	298 (5.22)	193.88 (3.53)
**Religion**		
Orthodox	1601 (28.06)	1840.91 (33.54)
Protestant	1048 (18.37)	1452.63 (26.47)
Muslim	2963 (51.93)	2098.60 (38.24)
Others	94 (1.65)	96.15 (1.75)
**Marital status**		
Single	30 (0.53)	22.44 (0.41)
Married	5438 (95.30)	5254.60 (95.74)
Divorced	59 (1.03)	65.58 (1.20)
Divorced	179 (3.14)	145.67 (2.65)
**ANC visits**		
No visit	2820 (49.42)	2604.44 (47.45)
1–2 visit	482 (8.45)	418.20 (7.62)
3 visit	756 (13.25)	790.43 (14.40)
≥ 4 visits	1648 (28.88)	11,675.23 (30.52)
**Place of residence**		
urban	1320 (23.13)	1361.00 (24.80)
rural	4386 (76.87)	4127.31 (75.20)
**Place of delivery**		
Home	2859 (50.1)	2821.85 (51.42)
Health facility	2847 (49.89)	2666.45 (48.58)
**Timing of ANC visit**		
< 3mths	108 (3.72)	85.22 (2.95)
3-6mths	2241 (77.28)	2302.83 (79.67)
7mths	3397 (59.53)	502.36 (17.38)
**Literacy**		
Can’t read at all sentence Read and write sentence	3632 (63.65) 568 (9.95)	3531.04 (64.34) 661.36 (12.05)
Read whole sentence	1116 (19.56)	1143.20 (20.83)
No card with required language.	390 (6.83)	152.71 (2.78)
**Wealth status**		
Poor	2932 (51.38)	2489.70 (45.53)
Middle	796 (13.95)	1037.39 (18.90)
Rich	1978 (34.67)	1952.21 (35.57)
**Birth order**		
First	1254 (21.98)	1196.83 (21.81)
2nd	1055 (18.48)	985.10 (17.95)
> 3	3397 (59.53)	3306.38 (60.24)
**Sex of the household head**		
Male	4560 (79.92)	4738.41 (86.34)
Female	1146 (20.08)	749.89 (13.66)

NB: *SNNP* South Nation Nationalities people, *ANC* Antenatal care

### Spatial analysis

#### Moran analysis

**Fig. 1:**
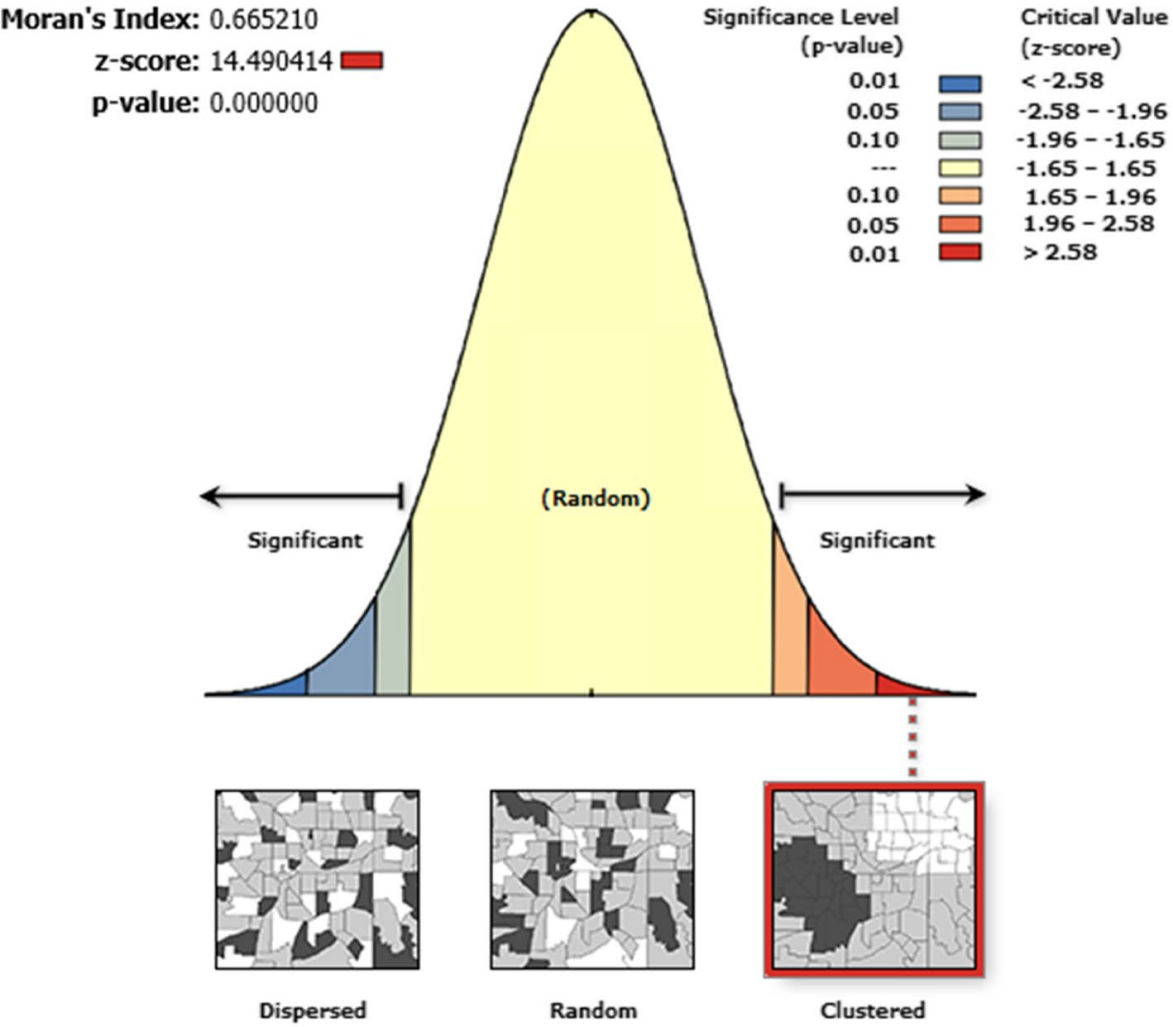
Spatial autocorrelation (Moran I) of institutional delivery in Ethiopia, EMDHS 201

Examining spatial autocorrelation, we recognized that the distribution of institutional delivery was non-random in Ethiopia. We also learned that the global Moran’s I test was 0.66(*p* < 0.0001). As global Moran’s I is significant and greater than zero, we concluded that the distribution was clustered. By this, the null hypothesis which stated random distribution of institutional delivery was rejected at Moran’s I test of 0.66(*p* < 0.001) (Fig. 1).

#### Spatial distribution of institutional delivery

**Fig. 2:**
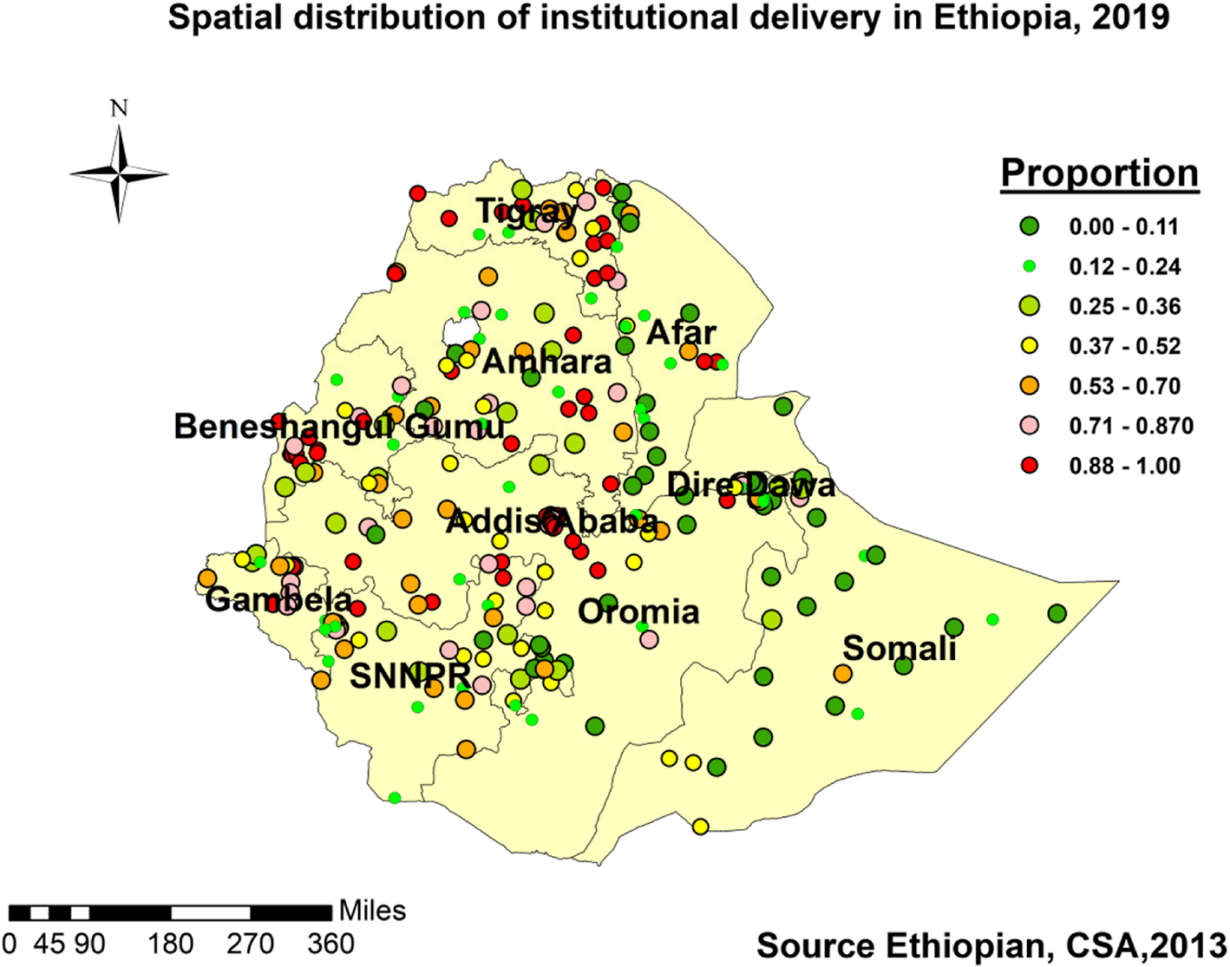
Spatial distribution of institutional delivery in Ethiopian, EMDHS 2019

The geo-distribution of institutional delivery was clustered in some parts of the country. Based on the Gettis-OrdGi statistical analysis, Addis Ababa city, Dire Dawa city, Hawassa town in SNNP, some places in Benishangul Gumuz, and few places in the Oromia region displayed the highest prevalence of institutional delivery showing a significant Z-score with 90% and above confidence levels (Fig. 2).

#### Ordinary kriging interpolation

**Fig. 3:**
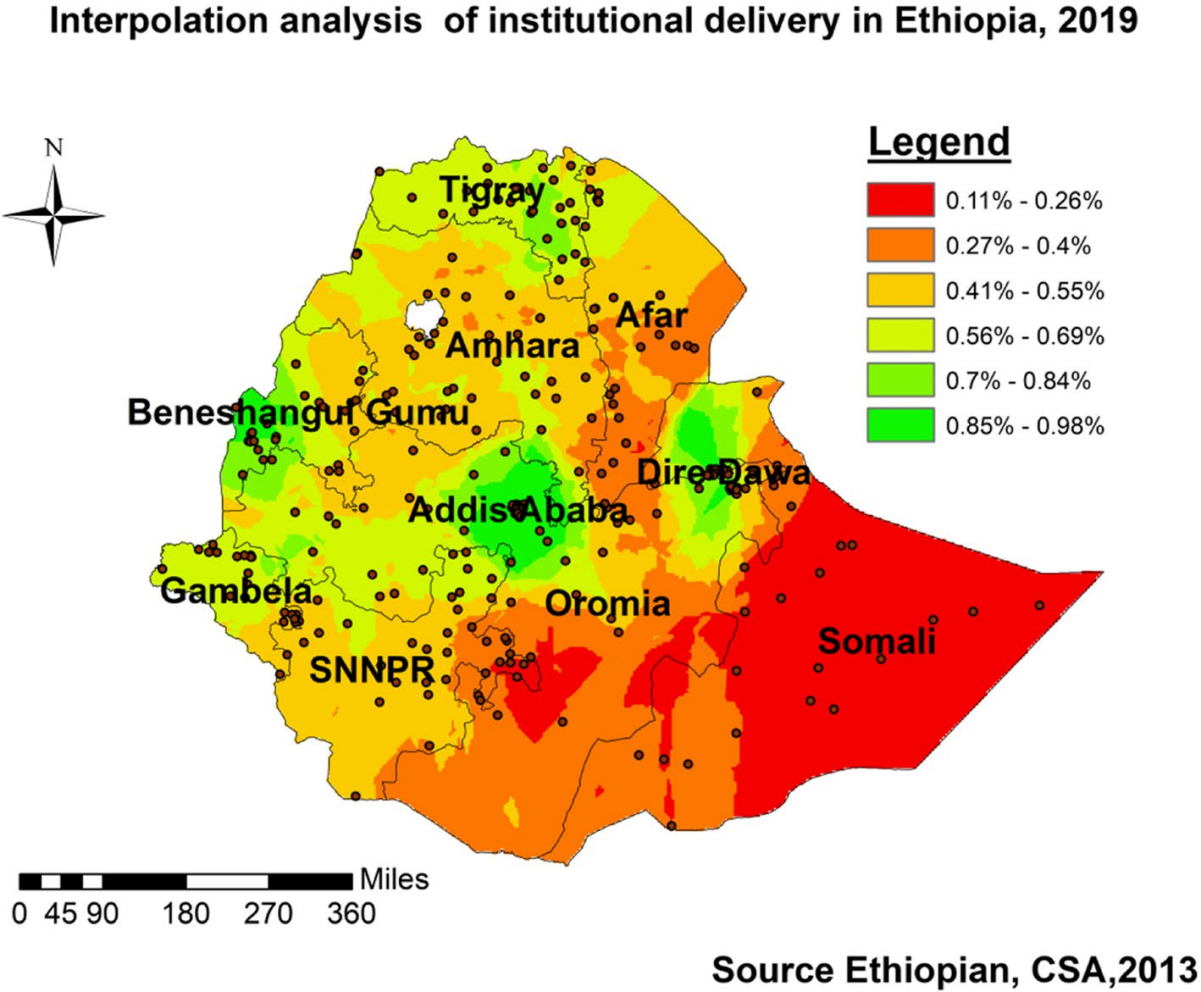
Ordinary Kriging interpolation of institutional delivery in Ethiopia, EMDHS 2019

To determine the occurrence of institutional delivery throughout the un-sampled areas, Ordinary Kriging interpolation measured the distance from the known point to predict unknown points/areas and indicated the point in the ranges of the event occurrences. The evidence from Fig. 3 showed that the possible areas for the event happening were Addis Ababa, Dire Dawa, Benishangule, Tigray, and some parts of Gambella.

#### SaTscan statistics

**Fig. 4:**
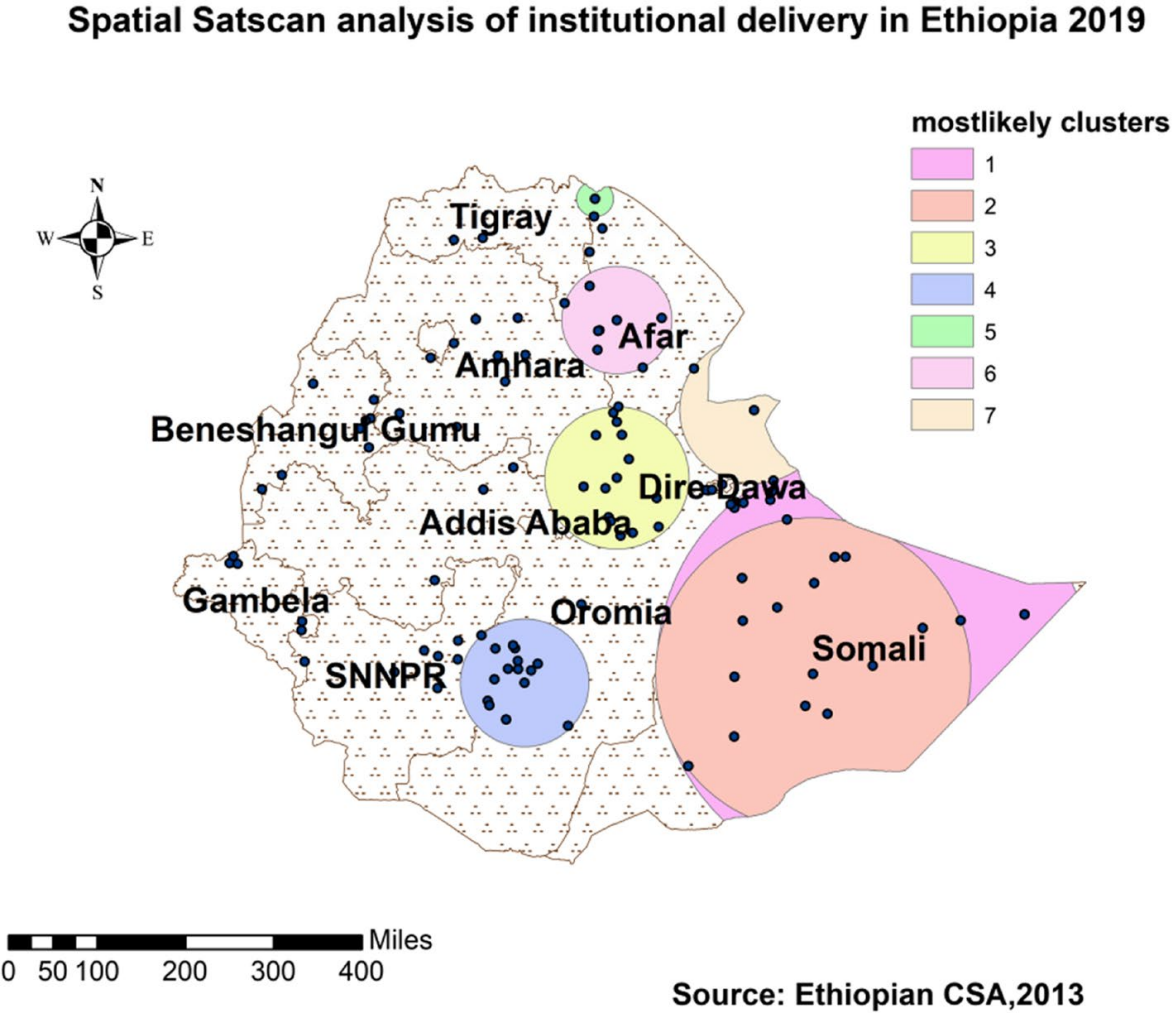
SaTScan scan statistics of institutional delivery in Ethiopia, EMHS 2019

In all spatial analyses above, we tried to identify the nature of institutional delivery distribution in the country; furthermore, SaTscan statistics enabled us to identify specific local clusters, which are necessary for specific local interventions. According to Table 2 and Fig. 4, there were one primary/most likely and six secondary and significant clusters in the country. Totally 27 locations with Coordinates/radius - (6.639662 N, 44.465853 E) / 390.28 km) with relative risk (RR) of 1.80 and LL of 187.82 were included in the primary cluster. The cluster was located in the Somali region including some borders of Oromia. It means these areas were 1.80 (*p* < 0.001) times more at risk of home delivery. The remaining clusters are secondary and presented in Table 2 and Fig. 4.

**Table 2:** Most likely clusters of institutional delivery among women of childbearing age in Ethiopia based on the EMDHS 2019

Clusters	Expected cases	Observed case	RR	LLR	***p***-value	Location regions
1	356.98	1.64	1.80	187.82	< 0.001	Somali and Oromia
2	217.27	1.69	1.79	129.88	< 0.001	Somali and Oromia
3	436.40	1.35	1.66	71.39	< 0.001	parts of Afar, Oromia, and Amhara
4	174.64	1.52	1.57	55.50	< 0.001	Eastern part of SNNP
5	30.31	1.91	1.93	34.56	< 0.001	Afar and Tigray
6	116.08	1.48	1.51	30.37	< 0.001	Afar, Amhara, and Tigray
7	36.98	1.78	1.80	27.94	< 0.001	Somali and Afar

#### Multilevel modeling of the institutional delivery

During multilevel modeling: age, highest education level achieved, preceding birth interval, literacy status, wealth status, and birth order at the individual level and place of residence and region at community level were significant associated with institutional delivery.

#### Individual-level

Women in the age group of 20–24, 25–29, and 30–34 years were 2.87, 2.70, and 2.92 times more likely to deliver in institutions with AOR of 2.87(1.02–8.10), 2.70(0.93–7.90), and 2.92(1.29–6.61) respectively. Women who learned primary, secondary and higher education had higher odds of delivering in institutions with AOR of 1.52(1.20–1.95), 1.77(1.03–3.07), and 5.41(1.91–15.25) respectively relative to those not educated. The odds of delivering in health institutions were also increased as the preceding birth interval increased with AOR of 1.01(1.01–1.02). Women who can read and write sentences had a higher tendency of delivering in health institutions with AOR of 1.94(1.28–2.94) unlike those who can’t. Rich women had higher odds of delivering in health institutions with AOR of 2.40(1.82–3.16). Birth order of second had higher odds to happen in institutions than the first birth with AOR of 1.86(1.41–2.44). Moreover, women who followed 1–2, 3, and ≥ 4 ANCs had higher odds of delivering in health institutions with AOR of 2.08(1.57–2.76), 3.24(2.51–418), and 4.91(3.93–6.15) respectively.

#### Community-level

Women in Tigray, Amhara, Benishangul, SNNP, Gambella, Harari, Addis Ababa, and Dire Dawa had higher odds of delivering in institutions compared to those in Afar with AOR of 9.35(3.83–22.90), 3.82(1.67–8.73), 13.00(5.89–28.69), 4.89(2.21–10.81), 4.22(1.75–10.17), 3.67(1.60–8.42), 13.15(3.22–53.57), and 4.83(2.05–11.38) respectively, while women in those regions who live in a rural area had 78% reduced chance of delivering in health institutions with AOR of 0.18(0.11–0.31) (Table 3).

**Table 3:** Multilevel modeling of institutional delivery among women aged 15-49 years in Ethiopia, 2019

Variables	Model 0	Model I	Model II	Model III
**Age**			–	
15–19	–	1	–	1
20–24	–	2.46 (1.10–5.52)^*^	–	2.38 (1.07–5.30)^*^
25–29	–	2.68 (1.20–5.97)^*^	–	2.422 (1.10–5.36)^*^
30–34	–	3.43 (1.51–7.7.9) ^**^	–	2.92 (1.29–6.61)^*^
35–39	–	3.13 (1.33–7.24) ^**^	–	2.60 (1.12–6.02)
40–44	–	3.08 (1.26–7.54)^*^	–	2.50 (1.30–6.10)
45–49	–	2.32 (0.85–6.27)	–	1.70 (0.62–4.52)
**Highest education level achieved**	–		–	
No education	–	1	–	1
Primary education	–	1.60 (1.25–2.06) ^**^	–	1.52 (1.20–1.95) ^**^
Secondary education	–	1.96 (1.13–3.39) ^**^	–	1.77 (1.03–3.07) ^**^
Higher education	–	6.89 (2.48–19.17)^**^	–	5.41 (1.91–15.25)^**^
Preceding birth interval	–	1.01 (1.00–1.02)^***^	–	1.01 (1.00–1.02)^***^
**Literacy status**	–		–	
Not read and write	–	1	–	1
Able to read whole sentence		1.24 (0.88–1.75)		1.15 (0.81–1.62)
Read and write sentence		2.19 (1.44–3.35)^*^		1.94 (1.28–2.94)^*^
No card with the language		1.32 (0.84–2.06)		1.51 (0.94–2.40)
**ANC visits**	–		–	
No ANC	–	1	–	1
1–2 ANC	–	2.09 (1.57–2.77)^***^	–	2.08 (1.57–2.76)^***^
3 ANC	–	3.27 (2.57–4.23) ^***^	–	3.24 (2.51–418) ^***^
≥ 4 ANCs	–	5.19 (4.15–6.48)^***^	–	4.91 (3.93–6.15)^***^
**Wealth status**	–		–	
Poor	–	1	–	1
Middle	–	1.10 (0.84–1.44)	–	1.06 (0.81–1.38)
Rich	–	3.48 (2.66–4.56)^***^	–	2.40 (1.82–3.16)^***^
**Birth order**	–		–	
First		Empty		Empty
2nd		1.80 (1.444–2.47)^***^		1.86 (1.41–2.44)^***^
> 3		Omitted		Omitted
**Region**	–			
Tigray	–		19.14 (7.70–47.56)^***^	9.35 (3.83–22.90) ^***^
Afar	–		1	1
Amhara	–		7.63 (3.33–17.58) ^***^	3.82 (1.67–8.73) ^***^
Oromia	–		4.11 (1.81–9.30) ^***^	2.39 (1.14–5.01)
Somali	–		0.53 (0.21–1.31)	0.93 (0.44–2.09)
Benishangul	–		18.29 (7.42–45.06)^***^	13.00 (5.89–28.69) ^***^
SNNPR	–		5.17 (2.29–11.70) ^***^	4.89 (2.21–10.81) ^***^
Gambela	–		6.45 (2.65–15.68) ^***^	4.22 (1.75–10.17) ^***^
Harari	–		8.93.(3.45–23.10) ^***^	3.67 (1.60–8.42) ^***^
Addis Ababa	–		16.20 (5.15–50.98) ^***^	13.15 (3.22–53.57) ^***^
Dire Dawa	–		8.83 (3.34–23.35) ^***^	4.83 (2.05–11.38) ^***^
**Residence**	–			
Urban	–		1	
Rural	–		0.06 (0.03–0.10)^***^	0.18 (0.11–0.31)^***^

*SNNP* South Nation Nationalities people, *ANC* Antenatal care

NB: * = *p* < 0.05, ** = *p* < 0.01, & *** = *p* < 0.001

The application of multilevel binary logistic regression was proved vital by abridging the ICC (the variation observed only due to the differences among clusters) from 61 to 28%. The remaining 28% unexplained inter-community variation can be reduced by including more other community-level factors in the model. The increased log-likelihood and decreased deviance were an indication of good model fitness (Table 4).

**Table 4:** Model comparison and random effect distribution institutional delivery among reductive age women in Ethiopia in 2019

Random effect model comparison	Model 0	Model 1	Model 2	Model 3
Community-level Variance	5.33	1.57	2.07	1.33
Inter-cluster correlation (ICC)	0.62	0.32	0.38	0.28
Log likelihood ratio (LLR)	− 2861	− 1057	− 2743	− 1021
Deviance	5722	2114	5486	2042
Proportional change in variance (PCV)	Ref	0.71	0.48	0.75
Media odds ratio (MOR)	9.02			

## Discussion

### Institutional delivery

Of the 5488 women involved in the current study, 48.58% were given births in health institutions. Evidence from other studies in the country unveiled institutional delivery was 71.7% in Dejen [33], 78.8% in Bair Dar [22], 38% in Mandura district [20], and 38.9% in 2016 EDHS [12]. There was a high variation from pocket studies but it showed good improvement from EDHS 2016. All the inconsistencies might be an indication of inconsistent interventions that need consideration during the next interventions (Table 1).

### Individual and community level characteristics

Although there was no adequate evidence to support, the average number of children was 4.03 ± 2.47 in the current study. In Mizan Tapi SNNP, 40% of the women had 3 to 4 children [34]. It was 1 to 4, 2 to 4, and 4 to 6 children in other studies in the country [15, 34, 35]. There were many inconsistencies, but our study might show the representative national figure. In other words, the average birth interval was 40.61 ± 26.30 months. Evidence indicated that 43% of women in Ethiopia had an average preceding birth interval of 24–48 months, and in East Africa, 82.1% had a preceding birth interval of ≥24moths [36]. In Ethiopia and even in the East of Africa, the average ranged between 24 and 48 months that is a good improvement over decades. However, the numbers of uneducated (53%) and economically poor (45.53%) women in this study were still very high. One study indicated that 79.2% of women in Ethiopia were uneducated [36] and resides in poor households [12, 13]. The consistent finding might be an indication of unsuccessful previous efforts and need more considerations recently. In the same way, a large proportion of women had no ANC follow-up (47.45%) and 64.34% of them were unable to read and write. Evidence from other studies also showed that 54% of the women giving birth in Ethiopia were unable to read and write [37] and 56% of them followed no ANC [38]. Overall, women’s literacy status and ANC follow-up have remained below par and invite more intervention. The fact that more than 75% of women residing in the rural parts of the country might also contribute to the above findings as indicated in similar studies [15, 37] (Table 1).

### Spatial distributions

The attempt of localizing the findings to the specific regions for enhanced interventions was successful; the distribution of institutional delivery could guide interventions as it showed clustering in some parts of the country. The significant Moran’s test of positive and greater than zero, the hot/cold spot areas identified with significant z-score, the predictions made from known spatial location of event occurrence (spatial interpolation) of ≥0.56%, and spatial scan statistics which encircled significant areas were enabled us to identify the regions needed more attention of improving institutional delivery in the country and were supported with other studies [14, 16]. From Table 2 and Figs. 1, 2, 3, and 4, we recognized the clustering nature of institutional delivery in Ethiopia. Accordingly, Addis Ababa, Hawassa, Dire Dawa, some parts of Benishangule Gumuz, and Oromia were found to be areas with a high proportion of institutional deliveries. However, Somali, Afar, some parts of SNNP, Oromia, and Amhara also showed low institutional delivery. This finding is also consistent with a systematic review done in the country [8], EDHS 2016 [4], spatial variation study in Northern Ethiopia, and EDHS 2005–2011 [38–40].

### Multilevel analysis of predictors

According to multilevel binary logistic regression analysis, women in the age group of 20–24, 25–29, and 30–34 years had higher odds of institutional delivery. Similar evidences were found in other studies in the country [37, 41]. The consistent information might indicate institutional delivery increased at the time women might take more responsibilities for their births in the middle ages of the childbearing period. In other words, women’s education and literacy were independent predictors of institutional delivery. This is a very global finding and common in most studies [35, 42, 43] and might indicate that improving mothers’ educational and literacy status might be very vital to robust institutional delivery. Mothers who had any ANC visits showed a high correlation of delivering in institutions. The finding was also supported with plenty of evidence from similar studies [26, 35, 42]. The exposure of mothers to health professional counseling might be a factor that spurred the utilization; however, it might also be because of they were the only mothers who had access. Additionally, mothers with rich wealth status had a higher tendency of delivering in institutions as is also the case in other studies [36, 44]. This might show that in addition to access, availability, and affordability could be considered during interventions. In this study, mothers tend to give birth at health institutions for later pregnancies which were also common in other studies [45] (Table 3).

Contextual analysis indicated that except Oromia, all agrarian regions and city administrations showed good institutional deliveries; however, pastoralists regions (Somali and Afar) showed high home deliveries; henceforth, the home delivery was also higher in rural areas. Other studies also supported this finding [4, 16]. During this analysis, we recognized many limitations. Among them, disproportionate sampling, using third party data, secondary nature of data, hierarchical nature of the data, lack of GPS coordinates for few records, and cross sectional nature of the study were some of the limitations. To handle disproportionate sampling, we applied weighting to the data before analysis. To deal with the third party nature of the data, we applied all the necessary protocols which are internationally standards to all countries and described all methods and manipulation of the data under method section. Due to the hierarchical nature of the data, we conducted multilevel binary logistic analysis. We also followed all standard methods to comprehend whether each captured data fit to the standard set by international DHS authorities. For the data without co-ordinates, we removed the records so that each event can be located on the test map.

## Conclusions

After 2016 EDHS, EMDHS was the first country representative data to make an impression about distribution and factors associated with institutional deliveries in Ethiopia. According to our analysis, although there was good progress from some previous evidence, institutional delivery was still low and requires enhanced further interventions. Factors that contributed to low prevalence were age, highest education level achieved, preceding birth interval, literacy status, wealth status, birth order, regions, and rural residences. The positive correlation of institutional delivery with educational/literacy status and ANC might invite further interventions in the area of awareness, access, and availability of the service. Regional disparities need specific interventions as pastoralist regions showed poor institutional delivery, while economic support for women in the rural area needs to be in the equation as the part of the further policy interventions.

## Data Availability

The data used in this study are the third-party data available from the Demographic and Health Survey (http://www.dhsprogram.com) and can be easily accessed by following the protocol indicated in the methods and materials section.

## Abbreviations

WHO: World Health Organization
EDHS: Demographic Health Survey
EMDHS: Ethiopian Demographic Health survey
ANC: Antenatal Care
AOR: Adjusted Odds Ratio
CI: Confidence Interval
SNNPR /SNNP: South Nations Nationalities people Region
EHNRI: Ethiopian Health Nutrition and Research Institute
NRERC: Review Board and the National Research Ethics Review Committee
ICC: Intra-Cluster Correlation
LL: Log likelihood
RR: Relative Risk
MOR: Median Odds Ratio
PCV: Proportional Change in Variance
MDG: Millennium Development Goals
PWMHC: Participation of Women in Monthly Health Conference
CSA: Central Statistics Agency
P: *P*-value
GPS: Global Positioning System
